# Anaesthesia for a biopsy of corpus callosum in patient with a recent intra-operative anaphylaxis to an unknown anaesthetic allergen: a case report

**DOI:** 10.1186/s12871-018-0629-y

**Published:** 2018-11-08

**Authors:** Ana Licina

**Affiliations:** Victoria, Australia

**Keywords:** Anaphylaxis, Neuro-anaesthesia, Airway management

## Abstract

**Background:**

Anaphylaxis in general anaesthesia occurs with a frequency of 1:5000-1:20000. This clinical summary reports on the use of an effective risk management strategy employing second line anaesthesia agents and alternative endotracheal intubation tools in a patient with a recent history of an intra-operative anaphylaxis to an unknown anaesthetic agent.

**Case presentation:**

A 71-year-old male presented for a repeat biopsy of corpus callosum 4 days following the cancellation of the procedure for a presumed anaphylactic reaction to an unknown anaesthetic agent.

During the repeat care episode, the decision was made to proceed based on the urgent need for tissue diagnosis to facilitate further treatment and lack of feasibility for more definitive identification of the causative agent(s). A consideration was made of the optimum ways to manage and mitigate risk in this setting.

The airway was managed using flexible endoscopic intubation in a spontaneously ventilating awake patient. Continuous remifentanil infusion was maintained throughout the case. Anaesthesia was maintained with sevoflurane at less than one MAC, with an uneventful completion of the biopsy of corpus callosum. All of the anaesthetic agents used during the prior care episode, with the exception of remifentanil, were avoided.

**Conclusion:**

In cases of an anaphylaxis to an unknown anaesthetic allergen, anaesthetic strategy consists of careful risk mitigation and deployment of second agent approaches. Awake flexible endoscopic intubation and remifentanil infusion are viable alternatives to standard induction techniques.

## Background

Anaphylactic, incorporating anaphylactoid, reactions occur with a frequency of 1:5000-1:20000 under anaesthesia with the commonest implicated agents being neuromuscular blocking agents (NMBA’s) and antibiotics [1]. In the recent National Audit Project 6 (NAP6) Report, Anaesthesia, Surgery and Life-threatening Allergic Reactions it was found that antibiotics were identified as the culprit by the review panel 1.4 times more frequently than NMBAs [2].

Under usual conditions, detailed history and timeline of the anaphylactic event form a cornerstone of the assessment in addition to the tryptase levels and skin testing. Skin prick testing and intradermal testing are usually performed 4-6 weeks after the event to allow for the re-accumulation of histamine in mast cell granules. Earlier testing can lead to false negative results and is therefore not recommended [3].

Management of patients who present for surgery with a history of anaphylaxis under anaesthesia without an elucidated agent can be particularly challenging. A thorough review of the available information, including the timeline of the event is essential, combined with risk management choices including consideration of second line induction and airway techniques, as well as alternative antibiotic prophylaxis if indicated.

Awake flexible endoscopic intubation is a well described commonly used airway technique indicated in cases of potential difficult intubation or ventilation.

One of the advantages of awake flexible endoscopic intubation is the lack of need for the use of neuromuscular blocking agents as there is a priority to secure the definitive airway while maintaining spontaneous ventilation. Remifentanil, is a member of the 4-anilidopiperidine class short acting potent opioid, which in lower continuous infusion doses can be used as an adjunct sedative agent to an awake flexible endoscopic intubation [4]. Another advantage of synthetic anilidopiperidine derivatives, such as remifentanil and fentanyl, is a lower tendency to cause anaphylaxis as compared to endogenous opioids such as morphine [5].

This is the first case description of clinical management of a patient, who presents.

with a history of an anaphylaxis to an unknown anaesthetic allergen for a repeat care episode. The patient has provided a written informed consent for the publication of this case report.

## Case description

A 71 year-old male presented for an urgent biopsy of a lesion of Corpus Callosum on a background of clinically diagnosed intra-operative anaphylaxis for the same procedure 4 days prior, which was abandoned. Symptoms of the initial presentation, which brought the patient under the care of the Neurosurgical team, included impaired ability to self-care and mild cognitive dysfunction. Surgical and Medical teams deemed the histo-pathological diagnosis essential due to the need for tailored subsequent medical management including potential urgent administration of chemotherapy and radiation.

The specialised allergy testing was not feasible due to the brief 4 day timeline proposed between the two procedures. Decision was made to proceed with surgery due to the urgent need for the identification of the nature of the lesion.

Both patient and the prior care episode were evaluated. Patient had a background of treated hypertension, with no other underlying illnesses. Prior to this, he had undergone uneventful surgical procedures and anaesthesia.

History, records of the procedure, and tests for mast cell mediators, were reviewed by the attending anaesthetist. During the first care episode patient was given a single dose of midazolam, followed by remifentanil and propofol total intravenous anaesthesia, rocuronium and cephazolin in quick succession. The laryngoscopic view was a Grade 3 Cormack and Lehane, with direct laryngoscopy utilising a Macintosh 4 Blade. CMAC® Blade 4 Videolaryngoscope demonstrated an equivalent Grade 3 Cormack and Lehane view. Following successful airway management, patient developed intractable hypotension, concomitant bronchospasm and was diagnosed with clinical anaphylactic episode. The patient was treated with an adrenaline infusion, procedure was abandoned and he was admitted with an endotracheal tube in situ to Intensive Care Unit where he recovered uneventfully. The patient re-presented 4 days later for a repeat procedure. Specific allergen testing was unavailable and deemed not to be feasible at this point in time.

During the repeat care episode, decision was made to secure the airway via an awake flexible endoscopic intubation prior to administering general anaesthesia. Airway was topicalised generously using a mix of 4% nebulised xylocaine, 10% local anaesthetic sprays to the back of the pharynx, and topical co-phenylcaine nasal spray. In addition to the above, Disposable devilbis atomiser was used to a total of 9 mg.kg^− 1^ of xylocaine. Remifentanil infusion at the dose of 0.05 mcg.kg^− 1^.min^− 1^ was used during the intubation and airway was secured uneventfully through the nose with a flexible Storz^tm^ 5.1 mm video fiber-optic bronchoscope. After connecting the breathing circuit, confirmation of CO2 was obtained and patient was administered 250 mg of thiopentone. Remifentanil infusion was increased to 0.2 mcg.kg^− 1^.min^− 1^ and maintained at this level through the case. Anaesthesia was maintained with Sevoflurane at a total dose of less than 1 MAC. For infection prophylaxis, 600 mg of clindamycin was administered. Procedure was completed uneventfully, patient extubated and taken to recovery. Using the above technique, the only medication in common between the two episodes of care was remifentanil.

## Discussion and conclusion

Conducting anaesthesia for a patient with an allergy to an unknown aetiologic agent anaesthetic is a rare clinical risk management challenge. Without a clear identification of the culprit agent(s), anaesthetic clinical care is approached through a risk minimisation strategy consisting of a review of the timeline of previous anaphylactic episode, avoidance of the agents more likely to have caused the reaction, use of second line agents and consideration of alternative airway techniques.

A literature search yielded a limited number of related case reports [6]. It is recommended that anaesthesia for patients is only attempted after delineating an accurate cause of anaphylaxis and/or a potentially severe life threatening immunologic reaction. In our case, clinical need for diagnosis within a certain time frame precluded a search for an accurate culprit agent. Cornerstone of management therefore lay on appropriate risk assessment and mitigation through use of second line agents. In addition, avoidance of general anaesthesia was considered during the case planning for repeat care episode, however an awake craniotomy was not a surgically viable option.

Anaphylaxis is an acute, potentially lethal, multi-system syndrome, resulting from sudden release of mast cell- and basophil-derived mediators into the circulation [7]. In the literature, the term anaphylaxis increasingly applies to Immunoglobulin E (IgE) dependent mechanisms, non-IgE-dependent immunologic mechanisms (formerly called anaphylactoid reactions) and nonimmunologic mechanisms (formerly called anaphylactoid reactions) involving direct release of histamine and other mediators from mast cells and basophils [7].

Confirmation of clinical diagnosis is based on the relative rise of serum tryptase. Skin-prick testing can assist with the diagnosis of IgE mediated reactions. In non-IgE mediated clinical spectrum of anaphylaxis, skin testing is negative. Serum tryptase in our patient post event was found to be 8 ng/ml, with a note that although this is not higher than the absolute tryptase level expected, it constituted a relative rise of 2 ng/ml from the baseline in this patient. It is thought that in patients who have a lower level of serum tryptase, a dynamic rise of tryptase from baseline level is indicative of mast cell activation [8]. It is however of note that the authors and immunologists in the latest National Audit Project (NAP6) reached a consensus on guidelines defining anaphylactic reactions. In this particular consensus definition, an essential component of diagnosing allergic anaphylaxis was the presence of sIgE in blood or skin, in addition to the other compulsory criteria of the event occurring within 60 min of induction, evidence of relative or absolute tryptase rise and the exclusion of differential criteria [8]. In cases such as ours, where skin and blood testing is not feasible due to the urgent timeline of medical care, such strict diagnostic criteria may preclude appropriate recognition and subsequent management of an episode of anaphylaxis. A broader clinical definition as outlined above and more prevalent in the literature which relies on a more inclusive scientific approach to anaphylaxis, may be more likely to facilitate safe post event management during repeat episodes of care.

There are various approaches to grading the severity of an episode of anaphylaxis as well as differential criteria delineating the grades ranging from one to four [9]. A helpful classification by Mertes was outlined in a recent paper defining the decision making process in the management of perioperative anaphylaxis [10]. In an attempt to clearly delineate each category of anaphylaxis, the authors of NAP6 have outlined their own modified definitions as outlined in the comparison Table 1. During the initial attempt at surgery, this patient had suffered a Grade 3 anaphylaxis with severe bronchospasm and hypotension, fitting both the NAP6 and Mertes classifications of anaphylaxis.

**Table 1:** Classification of severity of acute hypersensitivity reactions

Grade of reaction	Presence of symptoms Mertes et al	Presence of symptoms Cook et al
1	Presence of cutaneous signs	Rash, erythema, swelling (any of)
2	Presence of measurable but not life-threatening hypotension (defined as a decrease of more than 30% in blood pressure associated with unexplained tachycardia), difficulty of mechanical ventilation	Unexpected hypotension not severe (e.g. not requiring treatment), bronchospasm not severe (e.g. not requiring treatment), or both +/− Grade 1 features
3	Presence of life-threatening reactions, including profound hypotension (defined as a decrease of more than 50% of baseline), severe bronchospasm	Unexpected severe hypotension, and or severe bronchospasm, and or swelling with actual or potential airway compromise +/− Grade 1 features
4	Circulatory inefficacy (PEA arrest or arrhythmia), severe bronchospasm, inability to ventilate	Fulfilling indications for cardiopulmonary resuscitation
5	Grade 5 category not present in Mertes et al. classification.	Fatal

From Mertes et al., and Harper et al., *PEA* pulseless electrical activity

In this case, decision was made to abandon surgery immediately post the clinical diagnosis of anaphylaxis during the initial care episode, based on the preference of the surgeon and the anaesthetist at the time, in line with the more traditional teaching of limiting the complications of anaphylaxis [10].

Decision to proceed with surgery immediately following an episode of anaphylaxis is a multi-disciplinary decision based on the severity of the intra-operative crisis and the urgency of the procedural event. In a most recent report, this view is challenged in the light of the equivalent low level of complications in cases of where surgery was completed and in those where it was not [10]. As cancelling these cases can cause inefficiencies in healthcare, provided that anaphylaxis has been stabilised, it may be viable to complete the planned surgery provided it does not interfere with resuscitation due to anaphylaxis.

Due to the urgent clinical need for the diagnosis of the underlying medical condition in order to facilitate subsequent medical management, a repeat attempt at biopsy was required prior to the initiation of formal follow up skin testing.

Time line of the previous episode of anaphylaxis was reviewed, with the hypotension and tachycardia occurring within the 30 min of induction. It was therefore thought that one of the medications administered during the induction process was likely the causative agent. Patient had not been prepared or draped, therefore anti-septic agents were excluded from consideration as a potential cause of an anaphylactic reaction. In addition, all operating theaters at our institution have been deemed entirely latex free with no residual latex in any of the currently regularly used equipment, excluding urinary catheters. Latex allergy was therefore excluded from the differential diagnosis.

Antibiotics are reported as the commonest cause of peri-operative anaphylaxis in several series including United States, Australia, United Kingdom and Denmark [2].

In the most resent National Audit Project from the UK teicoplanin and co-amoxyclav alone were responsible for 89% of antibiotic related adverse events. In addition, in relation to timing in nearly all antibiotic-related anaphylactic episodes, infectious prophylaxis was administered prior to the induction of anaesthesia.

Our patient received cephazolin (a first generation cephalosporin) immediately following the induction agents but prior to intubation. As the various induction agents were administered in a close proximity with the antibiotic prophylaxis, clear delineation of aetiological agents based on the timeline of events was impossible.

Recommended timing of antibiotic administration at our institution, is guided by the prevention of Surgical Site Infection, with the current recommendations stating the antibiotics need to be administered within 30 min prior to the skin incision [11]. There is a strong argument toward guideline development to facilitate antibiotic prophylaxis at least 5-10 min prior to the induction of anaesthesia, allowing for the improvement in detecting an episode of anaphylaxis, and aiding investigation when needed [2].

Neuromuscular blocking agents are the commonest identifiable trigger of intra-operative anaphylaxis being responsible for 50-70% of reactions as reported in many European studies [12]. A’s cause anaphylaxis through both IgE and non-immunologic direct mast cell activation. Depolarizing agents are responsible for 21 % of anaphylactic episodes due to NMBA’s during anaesthesia [13]. Non-depolarizing agents are responsible for the remainder.

Alternative airway management through abolishing the spontaneous ventilation and direct laryngoscopy using suxamethonium had been considered during the case planning. The cross-reactivity between suxamethonium and non-depolarizing NMBA’s has been reported to be between 40 to 50% [9]. The risk of use of suxamethonium in this case was deemed too high based on the cross-reactivity figures between the depolarizing and non-depolarizing agents. Proactive risk management is part of an overarching principle of safety as a priority- with minimisation and avoidance of potential harmful behaviours [14].In this case avoidance of the use of a depolarizing agent with a high cross-reactivity potential with rocuronium was viewed as a risk mitigation strategy.

Awake flexible endoscopic intubation is a well described anaesthesia technique for the management of airway in patients in whom abolishing the spontaneous ventilation is undesirable or contra-indicated [15]. Complications of awake flexible endoscopic intubation include nose bleeds (10%), nodal rhythms (3%) and hypoxia (1.5%).

In addition, several case reports highlight the link between local anaesthetic administration for awake flexible endoscopic intubation and subsequent airway obstruction [16].

The failure rate of this technique has been reported at 1.5% in a retrospective study of 1612 sedated patients. Ability to perform awake fiber-optic intubation is considered a core component of anaesthetic competency by many professional anaesthesia colleges [16].

The indication for the use of awake flexible endoscopic intubation was dual- in addition to the desirability of the avoidance of use of NMBA’s, this patient had a history of being a Grade 3 Cormack and Lehane direct laryngoscopic view. One of the indications for the use of awake flexible endoscopic intubation is an anticipated difficult laryngoscopy and intubation in their own right, whether a patient may or may not be difficult to mask ventilate. Utilising a technique for a direct laryngoscopy without the adjunct neuromuscular blockers would have been possible, but presence of muscle tone would have likely made a Grade 3 Cormack and Lehane view more challenging therefore risking safety. The risk management decision was therefore made in favour of the awake flexible endoscopic intubation.

Awake rigid videolaryngoscopy in a spontaneous ventilating patient is an emerging approach to managing the difficult airway [17]. It has been argued that it may be a superior technique compared to the awake fiber-optic approach due to the more familiar laryngoscopic psychomotor skills. In this case, a Grade 3 laryngoscopic view was obtained with the use of a MAC4 C-MAC Stortz blade during a previous anaesthesia induction resulting in anaphylaxis. This approach was therefore considered prohibitive in terms of the probability of technical success.

The awake endoscopic flexible technique was therefore chosen as the spontaneous ventilation approach to the airway due to the operator experience and preference.

Sodium Thiopental, a short acting barbiturate is more likely to cause intraoperative anaphylaxis with variable frequency reports of 1/400-1/30000. In this situation it was deemed more suitable to use a second line agent such as sodium thiopental due to the potential risk of using propofol as an allergen.

In neuroanaesthesia, total intravenous technique with propofol is preferred due to the subsequent decrease in cerebral metabolic rate with a matched decrease in cerebral blood flow [18]. Volatile anaesthesia can be used during neurosurgical procedures as the direct vasodilatory effects of sevoflurane are offset by the decrease in cerebral metabolic rate provided end-tidal volatile concentration is maintained under 1 MAC.

Opioid phenylpiperidine derivatives have the lowest rates of association with anaphylaxis during anaesthesia. Synthetic opioids do not directly induce histamine release and the mechanism is thought to IgE mediated. In this scenario, although remifentanil had been used during the relevant first anaphylactic episode, a risk management decision was made based on the likelihood of relevant medication as an anaphylactic trigger agent.

We have illustrated the use of an alternative airway strategy together with the use of second line co-induction agents and intra-operative remifentanil infusion to achieve the purpose of the avoidance of first line anaesthetic agents as potential allergen triggers. In cases where incomplete peri-operative evaluation occurs after an anaphylactic event, risk management anaesthetic strategies employing second line medication and intubation tools are necessary in order to optimise safety.

Awake flexible endoscopic intubation is considered an essential skill in the practice of anaesthesia and in this case superseded the potential of awake video-laryngoscopy as a technique of choice in a patient with a known difficult laryngoscopic view.

After a careful analysis of anaphylactic timeline during a prior episode, alternative airway approaches with a concurrent use of anaesthetic medication with low allergenic potential are a viable alternative.

## Abbreviations

IgE: Immunoglobulin E
MAC: Minimum Alveolar Concentration
MAC4: Macintosh 4
NAP6: National Audit Project 6
NMBA: Neuromuscular Blocking Agents
PEA: Pulseless Electrical Activity
